# Automated Whole Brain Tractography Affects Preoperative Surgical Decision Making

**DOI:** 10.7759/cureus.1656

**Published:** 2017-09-06

**Authors:** Hesham Zakaria, Sameah Haider, Ian Lee

**Affiliations:** 1 Department of Neurological Surgery, Henry Ford Health System

**Keywords:** neurosurgical outcomes, automated whole brain tractography, neurosurgery, neuro-oncology, tractography, brain mri, glioblastoma, neuronavigation

## Abstract

Surgery in and around eloquent brain structures poses a technical challenge when the goal of surgery is maximal safe resection. Magnetic resonance imaging (MRI) has revolutionized the diagnosis and treatment of neurological disorders, but tractography still remains limited in terms of utility because of the requisite manual labor and time required combined with the high risk of bias and inaccuracy. Automated whole brain tractography (AWBT) has simplified this workflow, overcoming historical barriers, and allowing for integration into modern neuronavigation. However, current literature showing the usefulness of this new technology is limited. In this study, we aimed to illustrate the utility of AWBT during cranial surgery and its ability to affect presurgical and intraoperative clinical decision making. We performed a retrospective chart review of cases that underwent AWBT for one year from July 2016 to July 2017. All patients underwent conventional anatomic MRI with and without contrast sequences, in addition to diffusion tensor imaging (DTI) on a 3 Tesla MRI scanner (Ingenia 3.0T, Philips, Amsterdam NL). Post-hoc AWBT processing was performed on a separate workstation. Patients were subsequently grouped into those that had undergone either language or motor mapping and those that did not. We compared both sets of patients to see any differences in patient age, sex, laterality of surgery, depth of resection from cortical surface, and smallest distance between the lesion and adjacent eloquent white matter tracts. We identified illustrative cases which demonstrated the ability of AWBT to affect surgical decision making. In this single-center series, we identified 73 total patients who underwent AWBT for intracranial surgery, of which 28 patients underwent either speech or language mapping. When comparing mapping to non-mapping patients, we found no difference with respect to age, gender, laterality of surgery, or whether the surgery was a revision. The distance between the lesion and eloquent white matter tracts demonstrated a statistically significant difference between mapping and non-mapping patients, namely in the corticospinal tract (p < 0.0001), the superior longitudinal fasciculus (p < 0.0001), and the arcuate fasciculus (p < 0.004). Patients who underwent mapping were at equal risk for having a postoperative deficit (p = 0.772) but had an improved chance of recovery (p = 0.041) after surgery. We believe this phenomenon is related to increased awareness and avoidance of functional tissue during surgery, which occurs due to the combination of preoperatively identifying white matter tracts with AWBT and intraoperatively testing margins with mapping. We provide two illustrative cases that show the impact of AWBT on patient outcomes. In conclusion, AWBT is relatively simple to perform and provides vital information for surgeons about eloquent white matter tracts that can be used to help improve patient outcomes.

## Introduction

Techniques for identifying white matter tracts have existed for decades [1]; however, traditional (ie, manual) tractography has several limitations which hinder its routine application. With manual tractography, a fiber tract is localized first by selecting specific regions of interest (ROI) that are thought to be along the course of the intended white matter tract. These ROIs are defined by anatomical reference points, and it is from these regions that white matter fiber tracts are derived. Consequently, manual tractography is limited by its prolonged workflow, user bias, and anatomical distortions which may further compound human error [2]. This is problematic because the applicability of tractography for a neurosurgeon is to help identify essential white matter tracts that may be around a potentially resectable lesion, such as a tumor, which by its very nature will have distorted anatomy [3]. Given these limitations, white matter tractography has been utilized infrequently and with skepticism in general practice.

New technologies have recently emerged which seem to have overcome the intrinsic limitations of manual tractography. Advancements in automating tract-based analysis of diffusion tensor imaging (DTI) obtained with magnetic resonance imaging (MRI) have dramatically cut the labor and time required for tractography acquisition, as well as improved reliability and validity [4]. With the automated algorithm, diffusion tensor data from each voxel of a brain MRI are combined to extrapolate the major tract bundles--thereby identifying the theoretical end of fiber tracts by localizing the points of lowest anisotropy. Ultimately, this generates an accurate picture of major white matter tracts in the brain, an approach known formally as automated whole brain tractography (AWBT). AWBT stands in stark contrast to manual tractography, which is labor intensive, intrinsically bias prone, and can only capture one tract at a time between predetermined ROIs [5].

AWBT has the potential to garner widespread use with its ease of implementation and intuitive integration with stereotactic neuronavigation. We describe herein our case series of patients in which AWBT augmented our presurgical planning as well as intraoperative extent of resection. In addition, we also present two illustrative cases where AWBT was foundational to presurgical planning, surgical decisions, and ultimately patient outcomes.

## Case presentation

Under a previously approved institutional review board (IRB #9755) study designed to examine the outcomes in craniotomies for tumor resection, we retrospectively reviewed the most recent patients in whom AWBT was utilized. We identified 73 consecutive patients that underwent open surgical resection of an intracranial tumor using the Synaptive Brightmatter™ System (Synaptive Medical, Toronto, Canada) between July 2016 to July 2017. All patients underwent conventional anatomic MRI with and without contrast sequences in addition to DTI on a 3 Tesla MRI scanner (Ingenia 3.0T, Philips, Amsterdam NL). Post-hoc AWBT processing was performed on a separate PC workstation using BrightMatter software (Synaptive Medical, Toronto Canada). Final patient registration to the navigation system occurred immediately preoperatively and was confirmed again during surgery using anatomic landmarks. We divided patients into those that had undergone mapping (either language or motor) and those that did not. In both sets of patients, we reported patient age at the time of surgery, gender, specific procedure performed, laterality of the procedure, depth of resection from cortical surface, and the smallest distance between the lesion and directly adjacent eloquent white matter tracts. All mapping surgeries were performed by two board certified neurosurgeons who specialize in oncologic surgery and are experienced in mapping. In our institution, we routinely perform tractography for lesions directly abutting major white matter tracts (i.e., the corticospinal tract, the superior longitudinal fasciculus, and the arcuate fasciculus). Student’s unpaired t-test and Fisher’s exact test were used to compare variables when appropriate. Outcomes, including temporary and permanent neurological outcomes, were also reported based on in-depth chart review of individual hospital courses. A temporary deficit was defined as a change in the neurological examination that returned to preoperative baseline within three months. Any persistent changes from baseline, whether anticipated or unanticipated, were considered permanent.

Results

Of the 73 total patients, 28 underwent either speech or motor mapping with AWBT (Table 1) and 45 utilized AWBT alone (Table 2). Specific demographics and comparison statistics between each group can be found in Table 3. When comparing the two groups of patients, there was no statistically significant difference in age, gender, laterality of surgery, and whether the surgery was a revision. Patients that underwent mapping did not have lesions of different depths; however, they did have lesions that were significantly closer to specific white matter tracts, namely the corticospinal [p < 0.0001], superior longitudinal fasciculus [p < 0.0001], or arcuate fasciculus [p = 0.004]. This makes sense intuitively, as patients who require supplementary mapping are thought to have higher risk lesions that are in close proximity to eloquent areas, such as white matter tracts, and therefore benefit from the additional protection of mapping. While there was no statistical difference in neurological deficit after surgery between mapping and non-mapping cases (p = 0.772), patients who underwent mapping had a higher rate of postoperative recovery (p = 0.041). This finding is crucial as, despite the notion that these patients have higher risk surgeries, they are at no increased risk for postoperative deficits and are more likely to recover after a deficit. This further signifies that healthy perilesional tissue was not irreversibly damaged.

**Table 1:** Patients Who Underwent Speech and/or Motor Mapping WHO: World Health Organization CST: Corticospinal Tract SLF: Superior Longitudinal Fasciculus AF: ​Arcuate Fasciculus Oligo: Oligodendroglioma MM: Millimeters Pt: Patient

Pt #	Age	Sex	Side	Craniotomy	Revision Surgery	Speech Map	Motor Map	Depth of Resection (MM)	MM From CST	MM from SLF	MM from AF	New Postop Deficit	Deficit Recovery within 3 mo?	Histology	Major Morbidity
1	75	M	Left	Parietal	No	Yes	No	31	19.6	2	2	No		Glioblastoma	None
2	70	M	Right	Frontal	Yes	Yes	Yes	14.2	0	17.1	22.1	No		Metastatic Squamous Cell	None
3	45	F	Right	Fronto-parietal	Yes	No	Yes	32.7	0	11.3	13.2	Yes	Yes	Metastatic Poorly Differentiated Carcinoma	None
4	31	F	Right	Frontal	Yes	No	Yes	47.4	28.6	24.5	30.2	No		Anaplastic Astrocytoma	None
5	26	M	Right	Parietal	No	No	Yes	54.5	0	13	15	No		Glioblastoma	None
6	29	M	Right	Frontal	Yes	Yes	Yes	37.5	12.3	17.3	8.6	No		Oligo	None
7	26	M	Left	Fronto-temporal	No	Yes	Yes	40.6	0	0	0	Yes	No	Glioblastoma	None
8	36	M	Left	Frontal	Yes	Yes	Yes	60.5	8	0	5.3	No		Glioblastoma	None
9	53	F	Left	Temporal	Yes	Yes	No	28	24.3	0	0	Yes	Yes	Anaplastic Oligo	None
10	38	F	Right	Parietal	Yes	No	Yes	24.5	16.7	23.7	18.5	No		Oligo	None
11	67	F	Left	Parietal	No	No	Yes	68.3	5	0	0	No		Glioblastoma	None
12	55	M	Left	Fronto-parietal	Yes	Yes	Yes	35.1	0	0	0	No		Glioblastoma	None
13	26	F	Left	Parietal	No	Yes	Yes	48.6	0	0	0	No		Astrocytoma	None
14	65	M	Right	Parietal	No	No	Yes	57.8	0	0	16.6	Yes	No	Metastatic Poorly Differentiated Carcinoma	None
15	44	M	Right	Parietal	Yes	No	Yes	41.1	0	18.7	0	No		Glioblastoma	None
16	64	M	Right	Frontal	No	No	Yes	39	14.7	8.9	14	No		Glioblastoma	None
17	73	F	Left	Parietal	No	No	Yes	39.6	0	0	24.1	No		Radiation Necrosis	None
18	32	M	Right	Frontal	No	Yes	Yes	97	0	0	0	No		Glioblastoma	None
19	62	M	Right	Fronto-parietal	No	No	Yes	40.4	0	11.3	18.3	No		Glioblastoma	None
20	66	M	Right	Fronto-parietal	Yes	No	Yes	50.4	0	0	0	No		Glioblastoma	None
21	76	F	Left	Frontal	Yes	No	Yes	54.9	0	0	18.1	No		Glioblastoma	None
22	62	F	Left	Parietal	Yes	No	Yes	74.8	0	0	0	No		Glioblastoma	None
23	26	F	Left	Temporal-Parietal	No	Yes	Yes	41.3	41	11.4	0	Yes	Yes	Anaplastic Oligo	None
24	63	M	Left	Frontal	Yes	No	Yes	20.6	0	14.3	33.8	Yes	Yes	Anaplastic Astrocytoma	None
25	48	M	Right	Frontal	No	No	Yes	68.8	0	0	42	Yes	Yes	Glioblastoma	None
26	76	M	Left	Temporal	No	Yes	No	53.4	25	36.1	0	No		Glioblastoma	None
27	70	M	Right	Parietal	No	No	Yes	41.8	0	0	19.8	No		Glioblastoma	None
28	45	F	Right	Parietal	No	No	Yes	41.7	10.5	26.8	15	No		Glioblastoma	None

**Table 2:** Patients Who Did Not Undergo Mapping WHO: World Health Organization CST: Corticospinal Tract SLF: Superior Longitudinal Fasciculus AF: ​Arcuate Fasciculus Oligo: Oligodendroglioma MM: Millimeters Pt: Patient

Pt #	Age	Sex	Side	Craniotomy	Revision Surgery	Depth of Resection (MM)	MM From CST	MM from SLF	MM from AF	New Postop Deficit	Deficit Recovery within 3 mo?	Histology	Major Morbidity
1	55	M	Left	Parietal	Yes	12.2	30	15	15	No		Metastatic Squamous Cell	
2	45	M	Right	Parietal	No	76.4	36	0	0	Yes	No	Glioblastoma	Intracerebral hemorrhage
3	54	F	Left	Frontal	No	68	3.5	3	15	Yes	No	Glioblastoma	Intracerebral hemorrhage
4	46	M	Right	Fronto-temporal	No	37.1	18	8.6	10	No		Glioblastoma	
5	46	M	Left	Temporal-Parietal	No	51.2	8.3	11.4	9.8	No		Atypical Meningioma	
6	70	M	Left	Fronto-temporal	No	50.3	22	26.8	26.8	No		Meningioma	
7	36	F	Left	Temporal	No	40	25.7	20.8	13	No		Anaplastic Astrocytoma	
8	56	M	Left	Parietal	Yes	37.7	0	9.6	10	No		Reactive Changes	
9	55	M	Left	Parietal	Yes	52.3	34.5	10.7	13.9	No		Reactive Changes	
10	55	F	Left	Temporal	No	52.6	15.9	16.4	15.9	No		Glioblastoma	
11	74	M	Right	Temporal	No	34.3	36.2	28.2	28.2	No		Glioblastoma	
12	64	M	Bilateral	Bicoronal	No	48.8	70.1	44.5	83.4	Yes	No	Atypical Meningioma	Intracerebral hemorrhage
13	57	M	Left	Frontal	Yes	69.2	21.2	33.3	35.8	No		Glioblastoma	
14	63	F	Left	Temporal	No	22.3	30.5	20.3	16.4	No		Anaplastic Astrocytoma	
15	61	M	Left	Parietal	No	76.1	0	0	0	No		Glioblastoma	
16	69	M	Right	Suboccipital	No	49.2	10.6	60.9	53.4	No		Metastatic Adenocarcinoma	
17	55	M	Bilateral	Bicoronal	No	91.5	17.9	17.2	22.3	No		Metastatic Melanoma	
18	53	F	Left	Fronto-temporal	No	41.7	30.1	40.5	52.3	No		Meningioma	
19	48	M	Left	Temporal	No	40.3	36.1	36.6	26.4	No		Glioblastoma	
20	52	M	Right	Temporal	Yes	12.8	37	27.8	14.6	No		Glioblastoma	
21	71	F	Right	Frontal	No	73.5	15.1	0	37.3	No		Glioblastoma	
22	38	M	Left	Parietal	Yes	44.1	0	12.4	10.1	No		Astrocytoma	
23	54	M	Right	Temporal	Yes	32	0	11.2	0	No		Diffuse Large B-Cell Lymphoma	
24	48	F	Right	Temporal	No	56.8	0	14.8	0	Yes	No	Anaplastic Astrocytoma	
25	60	F	Left	Temporal	Yes	40.3	18.5	30.4	16.6	Yes	No	Gliosarcoma	
26	48	F	Left	Fronto-temporal	No	31.5	19	40.9	52.1	No		Meningioma	
27	55	F	Right	Fronto-temporal	No	16.9	13.3	16.9	18.7	Yes	Yes	Metastatic Adenocarcinoma	
28	56	M	Right	Temporal	Yes	31	14.5	43.2	11.7	No		Glioblastoma	
29	73	F	Left	Occipital	No	26.1	51.6	22.6	23.4	Yes	No	Glioblastoma	Intracerebral hemorrhage
30	57	M	Left	Parietal	No	49	17.2	13.1	18.7	Yes	Yes	Metastatic Small Cell Carcinoma	
31	45	F	Right	Temporal	No	44.3	42.7	17.8	32.5	No		Oligo	
32	54	M	Right	Parietal	Yes	35.7	36.7	22.6	10.9	No		Glioblastoma	
33	77	M	Right	Temporal	No	44.3	25	31	12.6	No		Glioblastoma	
34	55	M	Right	Temporal	No	38.5	36.7	35.7	0	No		Anaplastic Astrocytoma	
35	53	M	Left	Frontal	Yes	74.2	7.3	21.1	53.2	No		Glioblastoma	
36	63	F	Left	Frontal	No	37.8	47	9.4	62.1	No		Meningioma	
37	42	M	Right	Frontal	Yes	34.3	31	6.3	21	No		Oligo	
38	71	M	Left	Temporal	No	50.6	26.4	36	10.7	No		Glioblastoma	
39	55	M	Right	Temporal-Parietal	No	19.1	43.9	31.9	32.2	No		Meningioma	
40	46	M	Right	Occipital	Yes	40.9	59.8	29.8	18.9	No		Glioblastoma	
41	27	M	Right	Frontal	Yes	71.4	0	0	0	No		Glioblastoma	
42	58	F	Left	Frontal	No	31	41.4	15.7	24.8	No		Metastatic Poorly Differentiated Carcinoma	
43	86	M	Left	Occipital	No	84.5	24.2	36.4	16.6	Yes	No	Glioblastoma	
44	59	M	Right	Parietal	No	52.3	41.7	19.1	0	No		Glioblastoma	
45	60	F	Right	Occipital	Yes	43	45.4	32.4	37.3	No		Glioblastoma	

**Table 3:** Comparative Statistics Student’s unpaired t-test and Fisher’s exact test were used to compare variables when appropriate. * signifies statistically significant results, and include CST distance, SLF distance, arcuate fasciculus distance, and deficit recovery after surgery.​ CST: corticospinal tract; GBM: glioblastoma; SD: standard deviation; SLF: superior longitudinal fasciculus

	With Monitoring			Without Monitoring			
Age							
Mean (SD)	51.75	(17.78)		56.11	(11.23)		p=0.252
Median (Range)	54	(26-76)		55	(27-86)		
Sex (%)							
Male	17	(60.71%)		30	(66.67%)		p=0.624
Female	11	(39.29%)		15	(33.33%)		
Laterality (%)							
Left	13	(46.43%)		23	(51.11%)		p=0.631
Right	15	(53.57%)		20	(44.44%)		
Bicoronal	0	(0.00%)		2	(4.44%)		
Revision (%)	13	(46.43%)		15	(33.33%)		p=0.325
Speech Mapping (%)	11	(39.29%)					
Motor Mapping (%)	25	(89.29%)					
Both (%)	8	(28.57%)					
Depth of Resection (mm)							
Mean (SD)	45.91	(17.53)		45.94	(18.70)		p=0.995
Median (Range)	41.5	(14.2-97)		43	(12.2-91.5)		
CST Distance (mm)							
Mean (SD)	7.35	(11.28)		25.38	(17.03)		p<0.0001*
Median (Range)	0	(0-41)		25	(0-70.1)		
SLF Distance (mm)							
Mean (SD)	8.44	(10.49)		21.83	(13.84)		p<0.0001*
Median (Range)	1	(0-36.1)		20.3	(0-60.9)		
Arcuate Fasciculus Distance (mm)							
Mean (SD)	11.31	(12.03)		21.86	(18.46)		p<0.004*
Median (Range)	10.9	(0-42)		16.6	(0-83.4)		
Deficit (%)	7	(25.00%)		9	(20.00%)		p=0.772
Deficit Recovery (%)	6	(85.71%)		2	(22.22%)		p=0.041*
GBM (%)	17	(60.71%)		21	(46.67%)		p=0.336
Meningioma (%)	0	(0.00%)		7	(15.56%)		
Metastasis (%)	3	(10.71%)		6	(13.33%)		
Astrocytoma (%)	3	(10.71%)		5	(11.11%)		
Oligodendroglioma (%)	4	(14.29%)		2	(4.44%)		
Other (%)	1	(3.57%)		4	(8.89%)		

The clinical utility of AWBT is best illustrated through specific case examples. We present two patient vignettes that demonstrate the notable impact of AWBT on the ultimate treatment strategy, whether by affecting presurgical planning, intraoperative surgical decision making, and/or by helping localize white matter tracts.

Case 1

**Figure 1:**
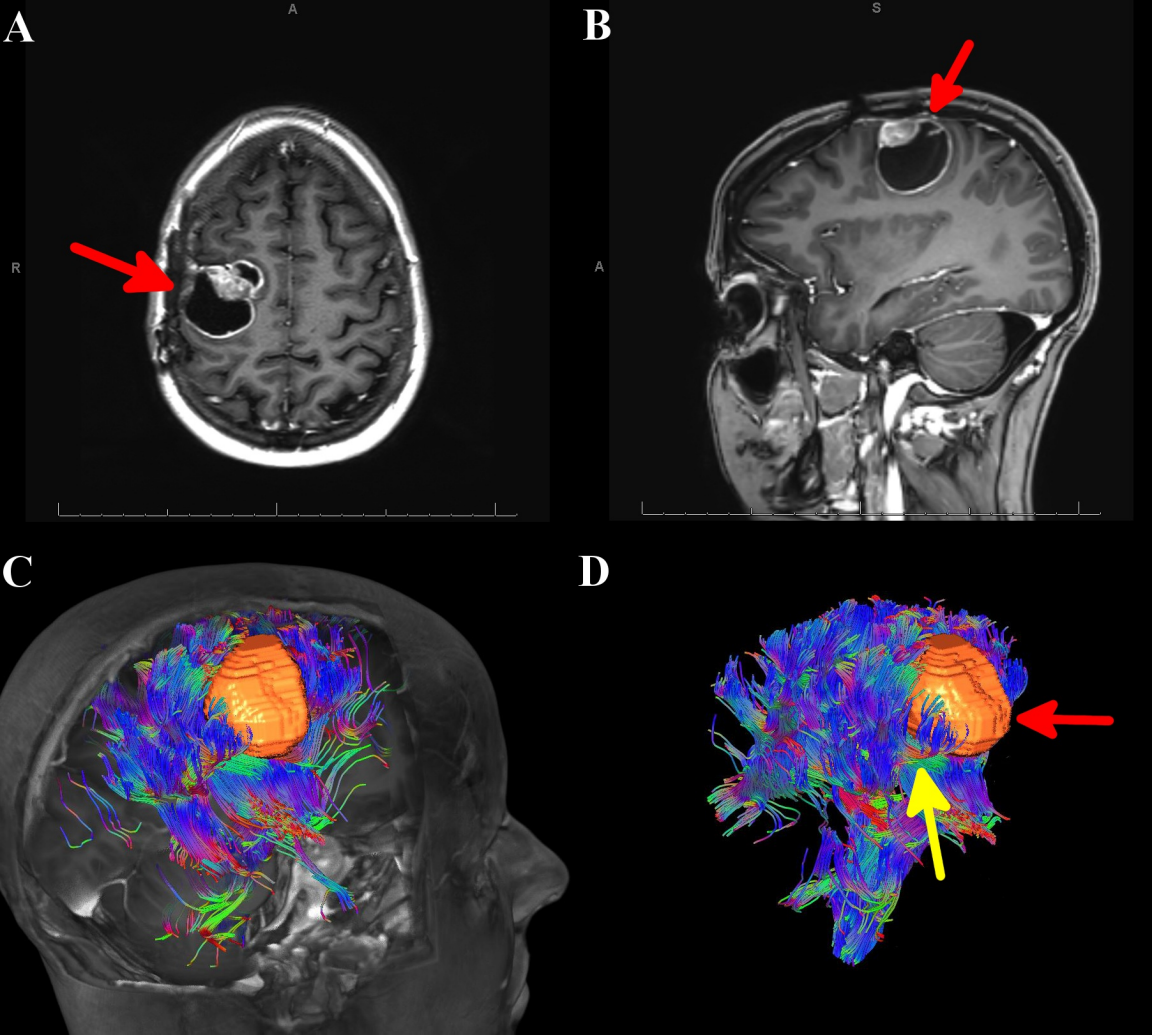
MRI of the brain and automated whole brain tractography (AWBT) for Case 1 A) Axial MRI of the brain with contrast showing a right-sided mass, denoted by a red arrow. B) Sagittal MRI of the brain with contrast showing a right-sided mass, denoted by a red arrow. C) AWBT image showing the tumor to be sitting within the corticospinal tract. D) AWBT image showing the tumor (denoted by red arrow), sitting within the corticospinal tract (denoted by yellow arrow). AWBT: automated whole brain tractography; MRI: magnetic resonance imaging

A 28-year-old female with a known history of anaplastic ependymoma (World Health Organization Grade III), which included two prior surgical resections and stereotactic radiosurgical treatment, presented with worsening left-hand paresis with loss of dexterity. An MRI of the brain with and without contrast demonstrated a 4-cm cystic lesion in the right frontal lobe with a focal nodule of enhancement at the medial border of the lesion (Figures 1A-1B). Calling on a priori knowledge of conventional sulcal anatomy, it was initially thought that this convexity lesion was displacing the corticospinal tracts (CSTs) posteriorly and medially. Since this was a superficial lesion, an anterior approach would be possible with caution being taken when dissecting at the lateral and posterior margins.

AWBT revealed that the lesion sat within and also divided the CSTs (Figures 1C-1D), and it was clear the previous surgical plan to enter the lesion anteriorly could jeopardize motor function. The surgical plan was modified whereby the lesion would be entered directly. The patient underwent an awake, right frontal craniotomy with intraoperative MRI and motor mapping, given that the AWBT demonstrated intimate proximity between the tumor and the CSTs. The reconstructed tractography data was fused with T1 MRI post-contrast imaging for stereotactic intraoperative neuronavigation. Cortical mapping was used to identify functional tissue as well as the boundaries of the lesion. Intraoperative mapping validated the tractography data - the lesion was embedded within the CST. Resection was undertaken in an en-bloc fashion with subsequent subcortical stimulation of the resection borders. In the deep posterior lateral border of the surgical cavity, left upper extremity motor responses were elicited consistently down to 4mAmp (Ojemann Cortical Stimulator, Integra Lifesciences, Plainsboro, NJ). This stimulation correlated well with tractography fused neuronavigation, again confirming the accuracy of AWBT. Postoperatively, the patient had no change in her neurological examination, despite surgery between the fibers of the CST. A postoperative MRI confirmed a gross total resection of the lesion. Pathology again revealed anaplastic ependymoma. Her hospital course was uneventful and she was discharged home on postoperative day 3. Over long-term follow-up, the patient remained neurologically unchanged.

Case 2

**Figure 2:**
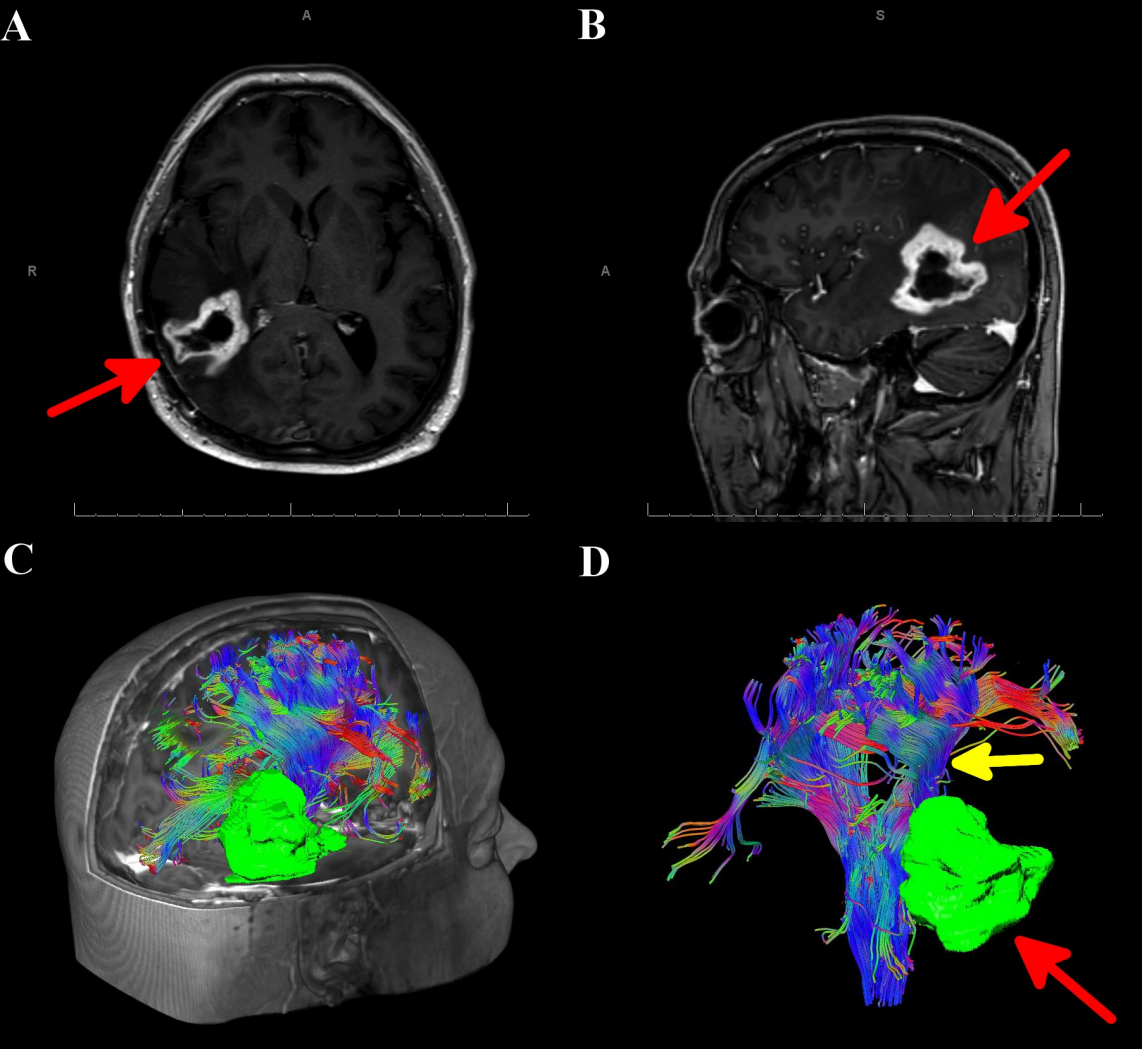
MRI of the brain and automated whole brain tractography(AWBT) for Case 2 A) Axial MRI of the brain with contrast showing a right-sided mass (denoted by red arrow). B) Sagittal MRI of the brain with contrast showing a right-sided mass (denoted by red arrow). C) AWBT image showing the tumor to be sitting deep and adjacent to the corticospinal tract. D) AWBT image showing the tumor (denoted by red arrow), sitting adjacent to the deep portions of the corticospinal tract (denoted by yellow arrow). AWBT: automated whole brain tractography; MRI: magnetic resonance imaging

A 44-year-old male with a known history of right parietal glioblastoma multiforme (World Health Organization Grade IV), which included post-subtotal resection, external beam radiation therapy, and adjuvant chemotherapy, presented to the clinic with an MRI demonstrating possible recurrence of disease (Figures 2A-2B). AWBT showed that the tumor was lateral and adjacent to the CSTs, which allowed for surgery using a lateral approach (Figures 2C-2D). AWBT showed close proximity of the descending corticospinal tract (CST) to the lesion, and the decision was made for the patient to undergo a right parietal craniotomy with intraoperative neurophysiological monitoring using motor-evoked potentials and subcortical stimulation. This was paramount for safe removal of the tumor during the deep and medial portion of the resection.

The stereotactic wand fused with AWBT was used to determine the optimal corticectomy site, and the tumor was removed in a piecemeal fashion via an intralesional approach. Upon approaching the deep margin of the tumor, subcortical stimulation was correlated with AWBT to localize the CST. Continued careful resection was undertaken until normal-appearing tissue was encountered. AWBT neuronavigation confirmed that the CST was deep to the resection cavity. This correlated accurately with subcortical stimulation of 10mAmp, implying a distance of at most 1 cm to the CST. Postoperatively, the patient maintained a stable neurological examination, with a postoperative MRI showing complete resection at the deep margins of the lesion adjacent to the CST. Pathology again showed glioblastoma multiforme. His hospital course was uneventful, and he was discharged home on postoperative day 2.

## Discussion

The goals of neuro-oncologic surgery are cytoreduction, tissue sampling, and reduction of mass effect, all while preserving functional perilesional brain. Unfortunately, in neurological surgery, the separation between tumor and functional tissue can be difficult to ascertain. This is particularly true with essential white matter tracts, such as the corticospinal tract (responsible for motor function) and the arcuate fasciculus (essential for language) [2]. It is only recently that AWBT has come to fruition, allowing these tracts to be reliably and accurately identified without bias and within a clinically applicable timespan [6]. This accomplishment has overcome a historical workflow limitation and made individualized tractography relatively easy to implement.

In this study, we show that patients who were selected to undergo mapping had lesions closer to essential white matter tracts, including the corticospinal tract, superior longitudinal fasciculus, and the arcuate fasciculus. We also found that while patients who underwent mapping had the same risk of postoperative deficit as non-mapping patients, they were more likely to have postoperative recovery of their neurological deficit. This is a counter-intuitive result, given that even though this population of patients had lesions that were closer to eloquent white matter tracts (and thus considered high risk), they had a better overall outcome with mapping than patients with lesions at low risk for iatrogenic injury. While it may be argued that patients who require mapping for tumors are a different surgical population than patients who do not, we think comparisons between these two populations are appropriate. These two populations of patients are naturally separated into ‘low-risk’ and ‘high-risk’ groups for iatrogenic injury, with mapping as an extra precaution for patients who are high-risk (their lesions were significantly closer to eloquent white matter structures). Our institute also has a traditionally low threshold for mapping, implying that the non-mapping patients were very unlikely to have an injury. Despite this, both populations had the same rate of postoperative deficit, and the high-risk population had only a transient deficit. This helps to illustrate that while AWBT is a useful adjunct, it cannot replace the gold standard of mapping for high-risk lesions intraoperatively; however, AWBT can be used to help decide which patients are more appropriate for mapping, and thus contributing to increased postoperative recovery by identifying patients who have lesions near previously unseen essential white matter tracts.

While AWBT is a significant improvement to manual tractography, it is still subject to the limitations inherent in all tractography. Peritumoral edema disrupts the fractional anisotropy that DTI uses to clarify white matter tracts. Crossing fibers may be difficult to isolate due to their low anisotropy [9] and intraoperative brain shift may provide inaccurate localization data [10].

Given our strong caseload of examples where AWBT affected surgical decision making by localizing previously invisible white matter tracts, combined with how easy it is to obtain, AWBT is now considered for all patients who are undergoing intracerebral tumor resection. For lesions near eloquent brain structures, AWBT has become the standard of care at our hospital. While our findings agree with those of comparable single-center series [7-8], prospective studies are necessary to show how AWBT can improve clinical decision making to further support its use as a mainstay for surgery in eloquent areas.

## Conclusions

While the current gold standard for localization of speech and language function remains direct cortical and subcortical stimulation, AWBT can provide critical preoperative and intraoperative information for the neurosurgeon. Our recent experience highlights the utility of AWBT in select cases of tumors near eloquent structures, and we provide specific examples of how AWBT changed the preoperative and intraoperative management of our surgical patients. Consequently, AWBT is now considered for all patients at our institution who are undergoing intracerebral tumor resection, regardless of whether we suspect the lesion to be near eloquent structures or not. Given the relative nascency of AWBT in tumor surgery, many questions regarding its role and applicability remain unanswered. Further studies are needed to determine the ideal patient population in whom to employ AWBT, what are the optimal tumor characteristics for AWBT, how peritumoral edema can affect AWBT accuracy, and does the supplemental use of AWBT have any effect on gross total resection or reoperation rates. The current body of evidence is largely predicated on case-control and cohort studies. In order to further delineate the role of AWBT in clinical practice, the neurosurgical community will benefit from prospective, randomized trials undertaken in well-defined patient populations.
